# Long-Term Performance of Nitrogen Removal and Microbial Analysis in an Anammox MBBR Reactor with Internal Circulation to Provide Low Concentration DO

**DOI:** 10.3390/toxics10110640

**Published:** 2022-10-25

**Authors:** Xuejiao Yin, Jiaxin Wen, Yihang Zhang, Xin Zhang, Jujiao Zhao

**Affiliations:** 1 School of Architecture and Engineering, Chongqing Industry Polytechnic College, Chongqing 401120, China; 2 Key Laboratory of the Three Gorges Reservoir Region’s Eco-Environment, Chongqing University, Chongqing 400045, China; 3 School of Environment and Resources, Chongqing Technology and Business University, Chongqing 400067, China

**Keywords:** anammox, nitrogen, MBBR, start-up, internal circulation

## Abstract

The anammox process is considered as a revolutionary new denitrification technology. In this study, the anammox process was started in a single-stage moving bed biofilm reactor (MBBR) and the mechanism of excess removal of ammonia nitrogen was studied. At stage I (day 0–51), anammox bacteria (AnAOB) was enriched by feeding synthetic sewage without adding organic carbon. The removal rate of ammonia nitrogen was maintained at about 54% and the removal rate of total inorganic nitrogen was maintained at about 62%. At stage II (day 52–91), internal circulation was added into the MBBR. After adding internal circulation, the ammonium removal efficiency reached about 96% (at day 56) and the total nitrogen removal efficiency reached about 86%. At day 90, the biofilm sample was drowned out for high-throughput sequencing. The results showed that the relative abundance of AnAOB was 23.23%. The dominant anammox genus was *Candidatus Brocadia*. The relative abundance of *Nitrosomonas* (ammonia oxidizing bacteria, AOB) was 0.63%. The excess ammonia nitrogen was removed by AOB and AnAOB through the partial nitrification and anammox (PNA) process.

## 1. Introduction

Anaerobic ammonium oxidizing bacteria (anammox) are capable of autotrophic ammonium oxidation with nitrite as an electron acceptor [1]. In recent years, the application of anammox technology in wastewater biological denitrification systems has been developing rapidly. At present, the anammox process has been successfully applied to the treatment of various nitrogen-containing wastewater such as landfill leachate, biogas slurry, pig manure wastewater, and aquaculture wastewater [2,3,4,5]. From the perspective of the environment and economy, compared with traditional nitrification and denitrification processes, the anammox process has the following advantages: low process cost and no need for additional organic carbon sources, thus saving energy and effectively reducing sludge production. The research shows that the application of the anammox process can reduce the aeration rate by 63% and sludge production by 80–90%. In addition, studies have shown that the application of the anammox process in the denitrification system can reduce the operational cost by 90% [6].

PN/A is the most commonly used wastewater denitrification process based on anammox [7]. In this process, AOB convert ammonium into nitrite and AnAOB convert the remaining ammonium and newly formed nitrite into nitrogen gas [8]. DO is one of the most important factors because DO can affect the growth of AOB and NOB (nitrite oxidizing bacteria). How to maintain a proper DO concentration to suppress the growth of NOB and promote the growth of AOB is one of the bottlenecks in the application of PN/A in actual sewage treatment projects [9]. Intermittent aeration has been proven to be an effective control strategy for the stable operation of the PN/A nitrogen removal system, which mainly uses the difference in oxygen affinity between AOB and NOB to complete the accumulation of nitrite in the system [8]. However, intermittent aeration lacks uniformity and stability. Under the long-term use of the aerator, it is possible for the microbial population to attach to it, thus affecting the stability of the oxygen supply of the reactor system. Therefore, it is urgent to develop a uniform and stable aeration mode.

The moving bed biofilm reactor (MBBR) using a biofilm carrier with a high surface area for biological retention has been proven to be an economically feasible nitrogen removal configuration. Compared with a traditional activated sludge reactor, MBBR requires a smaller reactor volume. MBBR has been widely used in wastewater treatment and achieved a good application effect. Kowalski et al. reported that the anammox process was successfully started in as little as 50 days in the MBBR [10].

In this study, a new stable and uniform mode to provide low concentration DO for an MBBR anammox reactor was developed. An internal circulation system was added to the MBBR. With the internal circulation system, the removal efficiency of ammonium increased greatly. Further study showed that the PN/A process dominated the excess removal of ammonium.

## 2. Materials and Methods

### 2.1. Reactor Design

**Figure 1:**
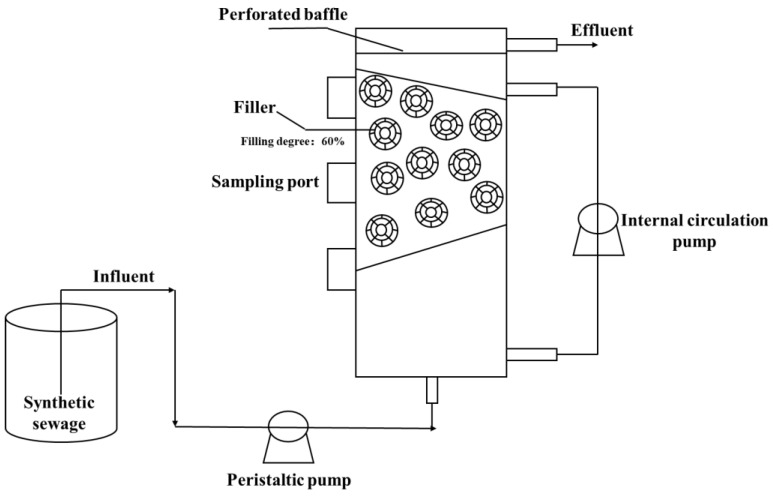
The schematic diagram of the MBBR.

The MBBR was made of plexiglass. The reactor was a cylindrical open reactor with a diameter of 150 mm and a height of 500 mm, and the effective volume was about 7.9 L. The reactor was filled with MBBR filler. The filling ratio of MBBR filler was appropriately set to about 60%. The effluent mode of the reactor was the overflow type. Untreated fresh synthetic sewage entered from the bottom of the reactor by a peristaltic pump and the same volume of sewage treated by the reactor flowed out from the top. The schematic diagram of the MBBR is shown in Figure 1.

### 2.2. Operation of the Reactor

Before start-up, the filler was filled to 60%, then the inoculated sludge and synthetic sewage were added, and finally the mixture was exposed to flow air to hang the film in the MBBR to make the sludge adhere to the filler. The aeration lasted for 12 h and the aeration flow was about 1.5 L/min. During aeration, air was introduced into the reactor from the bottom. After the completion of aeration, the manual water distribution operation was started. The operation of MBBR reactor after start-up was divided into two stages: the first stage (1–51 d), where the cycle period was 6 h, settling time was 20 min, the water inlet time was 10 min, and HRT = 12 h. In the second stage (52–91 d), the concentration of ammonia nitrogen and nitrite in the influent was kept unchanged, and the circulating pump was added into the system to promote the circulation of sediment and matrix in the system. The internal circulating flow was 0.3 L/min.

### 2.3. Synthetic Sewage, Filler, and Sludge

#### 2.3.1. Contents of Synthetic Sewage

The NH_4_^+^-N concentration in the influent was 140~180 mg/L and the nitrite concentration was 120–160 mg/L; The concentrations of other basic nutrients are shown in Table 1. Trace element I and II were configured as described by Van et al. [11].

#### 2.3.2. Filler

The filler used in this experiment was modified biological suspension filler (Dalian Yudu environment company, model wd-f25), which was made of high-density polyethylene, with a diameter and height of 25 mm and 10 mm, respectively, and relative density of 0.96–0.98 g/cm^3^. It had the advantages of a large specific surface area, easy film hanging, and good hydrophilicity. It had been widely used in various practical projects.

#### 2.3.3. Sludge

The activated sludge used in this experiment was from the Chongqing Ji Guanshi sewage treatment plant. The information of the Chongqing Ji Guanshi sewage treatment plant was introduced in a previous study [12]. The sludge of the reactor was taken from the anaerobic area. After inoculation, the initial MLSS of the reactor was about 2500 mg/L.

### 2.4. Chemical Analysis

Here, 50 mL supernatant samples were filtered before the analysis. The analyzed chemical index included ammonium, nitrite, and nitrate. The filtration was carried out on filter membranes (Jinteng, Tianjin, China) with a pore size of 0.45 μm. The ammonium, nitrate, and nitrite were analyzed three times in parallel by a colorimetric assay using a UV-2550 ultraviolet spectrophotometer (Shimadzu, Kyodo, Japan). The COD concentrations were detected by a COD analyzer (HACH, Loveland, CO, USA).

### 2.5. Microbial Analysis

#### 2.5.1. DNA Extract

Here, a 5 mL biofilm sample was withdrawn from the reactor and centrifuged at 8000 rpm for 5 min. Then, the supernatant was poured out. E.Z.N.A ^®^ Soil DNA Kit (omega bio TEK, Norcross, GA, USA) was used to extract microbial genomic DNA from samples according to instructions.

#### 2.5.2. Real-Time Quantification PCR (qPCR)

In this experiment, in order to obtain the abundance of denitrification-related bacteria, genomic DNA was extracted from 5 mL samples from the reactor, and nitrogen transformation genes including anammox 16S rRNA (amx 16S) gene, ammonia oxidation function gene (amoA), and denitrification function gene (NirS) were quantitatively analyzed by qPCR. The experimental process was as follows.

The total reaction system was 15 μL, which contained 7.5 μL SYBR Green premix (genstar, Beijing, China), 0.3 μL forward and reverse primers, 0.3 μL genomic DNA, and 6.6 μL ddH2O. The primer information of qPCR was as described in a previous study [13].

The R^2^ values of all standard curves were greater than 0.99. The CT value (threshold period) was determined to quantify the copy number of all of the studied genes and each reaction was repeated three times.

#### 2.5.3. High-Throughput Sequencing

The sludge samples in the reactor were sent to Majorbio Biopharm Technology Co, Ltd. (Shanghai, China) for high-throughput sequencing at the end of the second stage (90 day), resulting in 25,370 gene fragments with a base number of 10700706. The length of gene fragments was 262–433 bp. The primers used for high-throughput sequencing were bacterial universal primers 338f (5′-ACTCCTACGGAGGCAGCAG-3′) and 806r (5′-GGACTACHVGGTWTCTAAT-3′). Usearch was used for out statistics, while uparse (http://www.drive5.com/uparse/, accessed on 6 January 2020, version 7.0.1090) was used for out cluster analysis. RDP classifier Bayesian algorithm was used to classify 97% OTU representative sequences with a similar level, and the community species composition of each sample was counted. The comparison database was Silva (release132) http://www.arb-silva.de, accessed on 6 January 2020).

#### 2.5.4. Statistical Analysis

Statistical analyses were carried out using Microsoft Excel 2016 Analysis ToolPak. A statistical one-way ANOVA was applied to the results.

## 3. Results

### 3.1. Nitrogen Removal Performance

**Figure 2:**
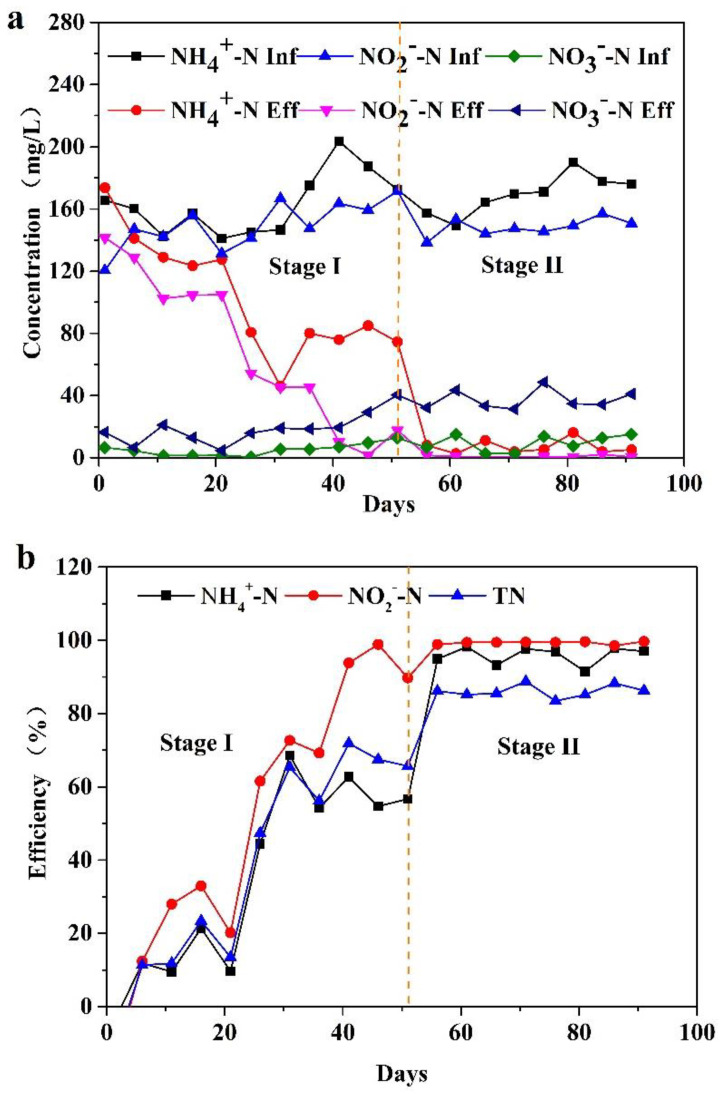
Concentrations (**a**) and removal efficiencies (**b**) of N-compounds in the influent and effluent of the reactor.

During the operation of the MBBR, the concentration changes in ammonia nitrogen, nitrite nitrogen, and nitrate nitrogen in the influent and effluent of the reactor were as shown in Figure 2. In the first stage, at the initial stage of operation, owing to the change in matrix, some unsuitable microorganisms in the reactor died and disintegrated, and some organic nitrogen was decomposed into ammonia nitrogen. Therefore, the contents of ammonia nitrogen and total inorganic nitrogen in the effluent increased on the first day of operation. The removal rate of ammonium and nitrite in the reactor increases gradually from the sixth day. From the 16th day, the removal of ammonia nitrogen and nitrite nitrogen in the system began to enter a stable state. By the 41st day, the removal of ammonia nitrogen and nitrite nitrogen reached about 128 mg/L and 153 mg/L, respectively; the removal efficiency of ammonia nitrogen and nitrite nitrogen reached 62.70% and 93.86%, respectively; and the removal rate of total inorganic nitrogen reached 71.83%. From day 41 to day 51, the removal of ammonia nitrogen and nitrite nitrogen in the MBBR was relatively stable. The removal rate of ammonia nitrogen was maintained at more than 54%, the removal rate of nitrite nitrogen was maintained at more than 89%, and the removal rate of total inorganic nitrogen was maintained at more than 62%. After adding the circulating pump in the second stage, the removal amount and removal efficiency of ammonia nitrogen in the MBBR were greatly improved, and the removal amount and removal rate of nitrite nitrogen did not change significantly. At this stage, the average removal amount of ammonia nitrogen reached 162.38 ± 11.06 mg/L and the average removal rate of ammonia nitrogen was 95.90% ± 2.45%. The average removal amount of nitrite nitrogen was 147.20 ± 5.28 mg/L and the average removal rate was 99.32% ± 0.39%. The average removal amount of total inorganic nitrogen was 281.73 ± 16.38 mg/L and the average removal rate was 86.11% ± 1.75%. From the above results, it can be seen that a good denitrification system was formed in the second stage in the MBBR.

### 3.2. Start up of the PN/A Process

**Figure 3:**
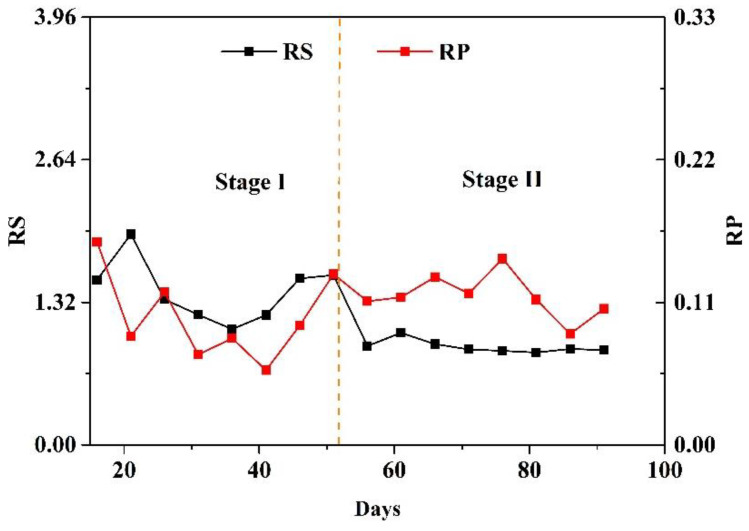
RS and RP value in the MBBR. Note: RS: consumption of nitrite/ammonium; RP: increase of nitrate/consumption of nitrite + ammonium.

Stoichiometric ratios can be used as indicators of the anammox process. The theoretical stoichiometric ratios of RS (consumption of nitrite to ammonium) and RP (production ratio of nitrate to consumption of the sum of ammonium and nitrite) of anammox reaction reported by Mulder et al. (1995) were 1.32 and 0.11, respectively. The RS and RP ratios in the MBBR in the first and second stages are shown in Figure 3. In the first stage, from 16 days to 51 days, the average value of RS in the MBBR was about 1.42 and the average value of RP was about 0.10. When only the anammox reaction occurs in the system, the theoretical value of RS was 1.32 and the theoretical value of RP was 0.11. The RS value of the first stage was slightly higher than that of the theoretical anammox reaction, while the RP value was slightly lower than that of the theoretical anammox reaction. This showed that, in the first stage, in addition to the anammox reaction, there was a small amount of denitrification in the MBBR. In the second stage, after adding the circulating pump, the average value of RS in the MBBR was about 0.91 and the average value of RP was about 0.11. In the second stage, the actual value of RS was lower than the theoretical value of RS for the anammox reaction. This showed that, in the second stage, in addition to the consumption of ammonia nitrogen by anammox, there were other reactions that consume ammonia nitrogen in the system. The ammonia consumption process might be partial nitrification or nitrification in the system. In addition, the actual value of RP in the MBBR reactor was equal to the theoretical value of RP in the anammox reaction and PN/A system. Based on the above results, it can be inferred that part of the PN/A denitrification system was formed in the MBBR at the second stage. Under the condition of constant influent ammonia nitrogen, the PN/A process completes the removal of residual ammonia nitrogen in the reactor. When the PN/A system began, the dissolved oxygen required for the partial nitrification process could be provided by increasing the internal circulation ratio instead of the aerator. In this study, there was a certain concentration of dissolved oxygen in the inlet water of the MBBR reactor. Under the action of an internal circulation pump, the matrix in the reactor can reach a state of uniform mixing, which may provide a suitable living environment for AOB.

### 3.3. Abundances of Nitrogen-Metabolism-Related Functional Genes in the MBBR

**Figure 4:**
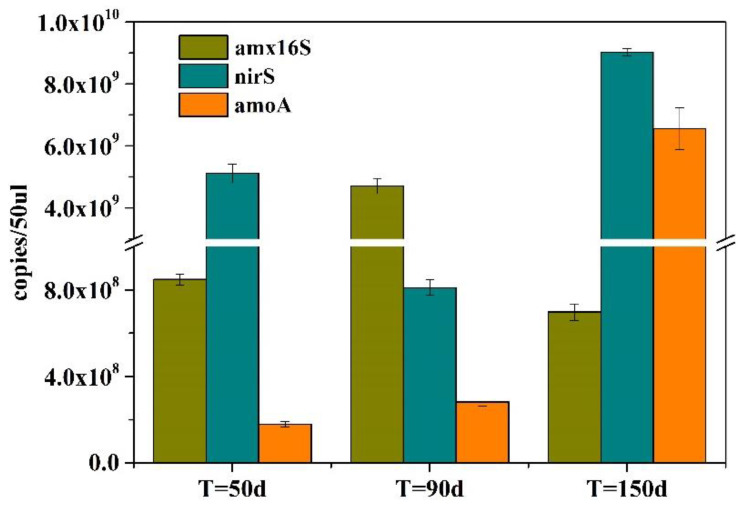
Abundances of nitrogen-metabolism-related functional genes in the MBBR at different operation times. amx16s: anammox 16s rRNA gene; nirS: nitrite reductase functional gene; amoA: ammonia oxidase functional gene.

During the MBBR operation, in order to determine the abundance of microorganisms and functional genes related to nitrogen transformation in the system and understand the impact of changes in culture conditions on microbial abundance, sludge samples were taken from the reactor for DNA extraction in the first stage (50 days) and the second stage (90 days), then the abundance of ammonia oxidizing functional gene amoA, denitrification functional gene Nirs, and anammox 16S rRNA genes in the samples were detected by absolute quantitative PCR; the qPCR test results are shown in Figure 4. In both stages, anaerobic ammonia oxidation 16S rRNA, denitrification function gene, and ammonia oxidation function gene were detected. In the first stage (50 days), the abundances of anaerobic ammonia oxidation 16S rRNA, denitrification function gene, and ammonia oxidation function gene were 8.48 × 10^8^, 5.11 × 10^9^, and 1.79 × 10^8^ copies/50 μL DNA samples, respectively. In the second stage (90 days), the abundances of anaerobic ammonia oxidation 16S rRNA, denitrification function gene, and ammonia oxidation function gene were 4.70 × 10^9^, 8.10 × 10^8^, and 2.82 × 10^8^ copies/50 μL DNA samples, respectively. Compared with the first stage, the copy number of anaerobic anammox 16S rRNA gene in the second stage in the MBBR reactor increased significantly. This showed that, in the second stage, although the internal circulation pump was added to supplement a small amount of dissolved oxygen, the environment in the system was still suitable for the growth of AnAOB, which continued to proliferate under the continuous supplement of reaction substrate and an appropriate growth environment. In the second stage, the copy number of denitrifying functional genes in the MBBR reactor decreased significantly, which may be due to the increase in the dissolved oxygen content and the lack of an organic carbon source in the matrix, which led to the inhibition of denitrifying bacteria activity and the significant reduction in abundance. In addition, the copy number of ammonia oxidation function gene in the second stage increased slightly, which may be due to a certain amount of proliferation of ammonia oxidation bacteria because of a small amount of dissolved oxygen.

### 3.4. Microbial Composition in the Reactor

#### 3.4.1. Phylogenetic Tree

**Figure 5:**
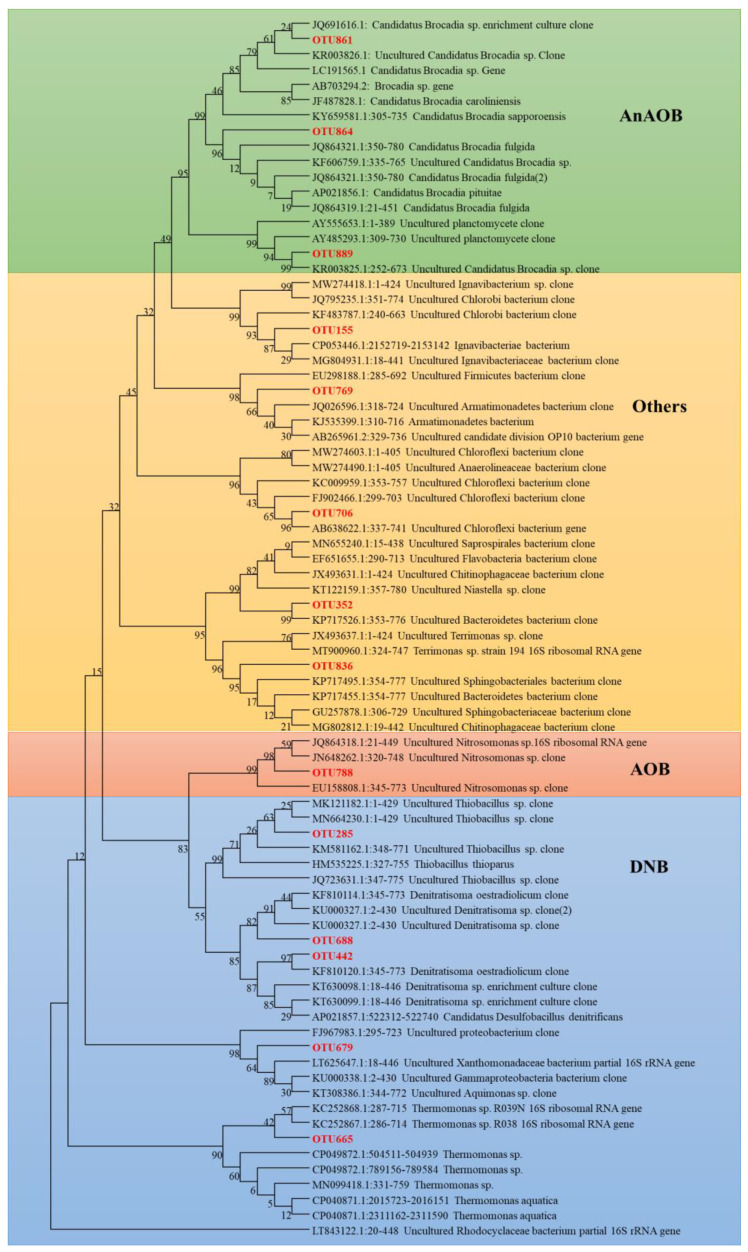
Phylogenetic tree of 14 dominant 16S rDNA partial sequences recovered from the MBBR reactor. 16S rDNA partial sequences recovered from this study are shown in red and closely related genes downloaded from the NCBI are shown in black. GenBank accession numbers of genes downloaded from the NCBI were presented at the beginning of these genes. Bootstrap support values are represented at branch nodes.

After high-throughput sequencing, 14 dominant 16S rDNA partial sequences were blasted on NCBI. According to the blasting results, a phylogenetic tree showing the subordinate relationship of the 16S rDNA gene sequences of bacteria in the MBBR reactor was constructed, as shown in Figure 5. The results showed that three OTUs belonged to AnAOB and had a close relationship with *Candidatus Brocadia*. Of which, OTU 861 had 99% similarity *Candidatus Brocadia caroliniensis* and OTU 864 had 99% similarity with *Candidatus Brocadia fulgida*. One OTU belonged to AOB and had 100% similarity with *Nitrosomonas* sp. Five OTUs belonged to denitrification bacteria (DNB). Among the five OTUs, OTU 285 was close to *Thiobacillus thioparus*, OTU 688 was close to *Denitratisoma oestradiolicum*, OTU 442 was close to *Denitratisoma oestradiolicum* either, OTU 679 was close to *Xanthomonadaceae*, and OTU 665 was close to *Thermomonas aquatic.* Seven OTUs belonged to bacteria other than AOB, AnAOB, and DNB.

#### 3.4.2. Abundances of Bacteria in the Reactor

**Figure 6:**
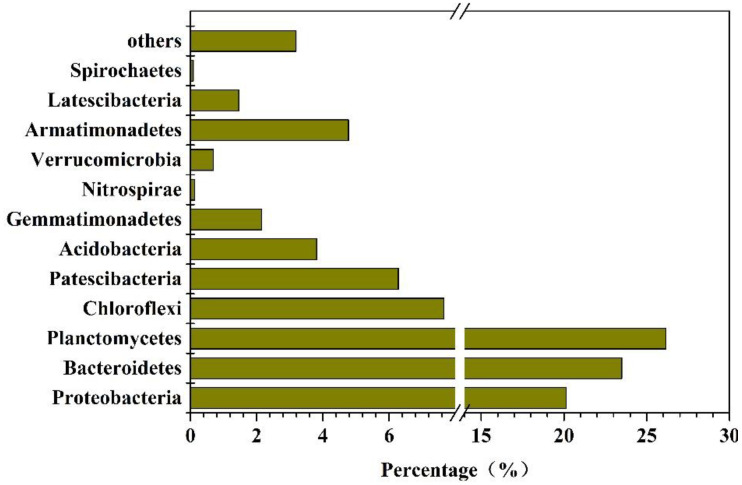
Bacterial abundances in the MBBR on the phylum level.

Because the denitrification effect of the second stage reactor was good and stable, a joint denitrification system of anammox and PN/A was formed. In order to understand the microbial composition and microbial action mechanism in the system at this stage, sludge samples were taken from the reactor for high-throughput sequencing at this stage (90d). After high-throughput sequencing and analysis, the composition of microorganisms in the system was determined as shown in Figure 6. The results of high-throughput sequencing showed that, on the phylum level, the microbial categories in the MBBR mainly included *Proteobacteria*, *Bacteroidetes*, *planctomycetes*, *Chloroflexi*, *Patescibacteria*, *Acidobacteria*, *Gemmatimonades*, *Nitrospirae*, *Verrucomicrobia*, *Armimonades*, *Latescibacteria,* and *Spirochaetes.* The total proportion of these microorganisms reached more than 96% of the total microbial abundance. Among them, *Planctomycetes*, to which AnAOB belongs, was the most significant, with a relative abundance of 26.6%. The relative abundance of *Nitrospirae* belonging to AOB was 0.13% and those of *Proteobacteria* and *Bacteroidetes* belonging to DNB were 20.14% and 23.49%, respectively. The relative abundance of *Chloroflex* with partial denitrification ability was 7.66%.

The levels related to nitrogen metabolism, microbial categories, and contents in the MBBR are shown in Table 2. According to Table 2, the total content of DNB in the MBBR in the second stage was 9.60%. Among them, *Denitratima* was the most significant, with a relative abundance of 3.76%, accounting for 39% of the total DNB content. The second was *Terrimonas*, with a relative abundance of 1.97%. The relative abundance of *Thermomonas* was also close to 1%. The reason the abundances of DNB in the MBBR was still considerable may be that the death and disintegration of some microorganisms in the reactor provides the energy required for the proliferation of some heterotrophic bacteria, such as DNB, and there was no substrate that can inhibit DNB in the reactor. The hypoxic environment in the reactor was more suitable for the survival of DNB. In addition, the most significant DNB in the reactor was *Denitratisoma*.

The total content of AOB and NOB in the second stage reactor was 9.60%. The AOB species was *Nitrosomonas* and its relative abundance was 0.63%. The species of NOB was *Nitrospira* and its relative abundance was 0.11%. *Nitrosomonadaceae* and *Omnitrophicaeota* were not detected in the MBBR, indicating that the environment in the MBBR was not suitable for the growth of these two bacteria. In addition, the relative abundance of *Nitrosomonas* in the reactor was higher than that of *Nitrospira*, which means that the low dissolved oxygen culture condition of the MBBR in the second stage was more suitable for the growth of nitrobacteria, which was conducive to the accumulation of nitrite. Ma et al. also showed that AOB had a stronger affinity for oxygen than NOB under the condition of a low concentration of dissolved oxygen [14]. At present, it was difficult to inhibit the activity of NOB during the start-up of the PN/A system, making it difficult to accumulate nitrite, so as to provide substrate for AnAOB, which was the biggest challenge faced in the application of the PN/A system in the mainstream anaerobic ammonia oxidation process [8].

In the second stage, the relative abundance of AnAOB in the MBBR was 23.23%. The reason AnAOB was enriched in the MBBR was that the matrix contained nitrite, which directly provides metabolic substrate for AnAOB. At the same time, the influent of the MBBR reactor did not contain organic carbon and the content of dissolved oxygen was low. These conditions made the MBBR very suitable for the growth of AnAOB. The AnAOB type in the MBBR was *Candidatus Brocadia*.

## 4. Discussion

Owing to the lack of nitrite in urban domestic sewage, the anammox process cannot be realized directly. Usually, partial nitrification and the anammox process are combined to complete nitrogen removal in waste water treatment plants (WWTPs) [15]. However, the long start-up time of the anammox process and the harsh growth environment for AOB make it hard to start the PN/A process and limit the application of the PN/A process in mainstream WWTPs [16]. Studies show that dissolved oxygen and temperature are the key factors affecting the enrichment of AOB [17,18,19]. When the temperature is higher than 25 °C, the specific growth rate of AOB is higher than that of nitrite oxidizing bacteria (NOB). When the temperature is below 15 °C, the maximum growth rate of AOB is lower than that of NOB, so it is impossible for AOB to outcompete NOB [17]. Lotti et al. reported that the activity of AnAOB decreased 10 times when the temperature was reduced from 30 °C to 10 °C. Therefore, temperature control is very important for the startup and stability of the PN/A system [18]. In terms of DO, one of the main strategies to suppress NOB is to control the oxygen struggle between AOB and NOB under the condition of limiting dissolved oxygen. Gilbert et al. showed that the medium content of DO (<1.0 mg/L) and low content of DO (<0.5 mg/L) were beneficial to the growth of *Nitrosomonas* (AOB) and could inhibit the growth of NOB [19,20]. The research of Daims et al. shows that, when the DO concentration is about 1.5~0.5 mg/L, NOB can still be inhibited with the coexistence of and change in the relative abundance of nitrifying bacteria and nitrifying spirochetes [21]. In a word, how to maintain the DO concentration at the proper level to enrich AOB and inhibit NOB is still a challenge in the PN/A application area.

In this study, the single-stage nitrogen removal system in the MBBR involves AOB, NOB, DB, AnAOB, and other microbial populations. DO was a key influencing factor for AnAOB, AOB, NOB, and DNB. Studies had shown that AnAOB were obligate anaerobic bacteria, so they are very sensitive to the presence of oxygen. An early study showed that AnAOB activity was completely inhibited at an oxygen concentration of 0.04 mg/L (0.5% of air saturation). However, the inhibition of DO varies depending on the type of AnAOB system used in the enrichment process [6]. DNB were facultative anaerobic bacteria and a too high DO content will also inhibit the activity of DNB. AOB was an aerobic microorganism and a low DO content will inhibit the activity of AOB [22]. Therefore, it was very important to control the content of DO when starting the PN/A system [9]. In this study, the PN/A system used the MBBR with packing, which will cause certain mass transfer resistance. Therefore, the content of dissolved oxygen inside and outside the filler will be different. In addition, owing to the formation of biofilm in the filler, a dissolved oxygen gradient will also be formed. The inner layer of the packing will form a hypoxic environment suitable for the growth of AnAOB and DNB, while the outer layer of the packing was an aerobic environment suitable for the growth of AOB and NOB. Therefore, the aeration volume in the system should not be too large, otherwise the DO content will be too high, thus inhibiting the activities of AnAOB. At the same time, the DO content should not be too low. Too low of a DO content will cause the activity of AOB to be inhibited, so ammonia nitrogen in influent cannot be transformed to provide reaction substrate for AnAOB. A low DO content will also lead to the growth of filamentous bacteria and affect the effluent quality [9]. In this study, before adding the internal circulation system, only the anammox reaction occurred in the reactor. After adding the internal circulation system to provide a small amount of DO, both the ammonia oxidizing reaction and anammox reaction occurred in the MBBR and the PN/A system was successfully started. It is worthwhile to notice that, with the internal circulating flow of 0.3 L/min, AOB outcompeted NOB and the nitrification reaction was successfully inhibited. The results indicated that adding an internal circulation system in the MBBR was a feasible strategy to start the PN/A process and could be a reference for the application of the PN/A process in actual sewage treatment projects.

## 5. Conclusions

The study showed that the PN/A denitrification system could be formed by adding the internal circulation system to the anammox MBBR. The PN/A process contributed to the excess removal of ammonia nitrogen. With the PN/A process, the ammonium removal efficiency in the anammox reactor reached up to 96% from 62.7%, while the total nitrogen removal efficiency reached about 86%. Under the internal circulation situation, the abundance of AOB (*Nitrosomonas*) outcompeted NOB (*Denitratisoma*). This study solved the problem that AOB was outcompeted by NOB, which limited the use of the PN/A process in practical wastewater treatment projects. However, the application of this research result in actual sewage treatment needs to be further verified in the pilot-scale reactor.

## Figures and Tables

**Table 1:** Concentrations of synthetic sewage contents.

Content	Concentration
NaHCO_3_	500.0 mg/L
KH_2_PO_4_	27.2 mg/L
MgSO_4_·7H_2_O	300.0 mg/L
CaC1_2_·2H_2_O	180.0 mg/L
Trace element I	1 mL/L
Trace element II	1 mL/L

**Table 2:** Nitrogen-metabolism-related bacteria and their abundances in the MBBR.

Genera	90 d (%)	Genera	90 d (%)
**DNB**		**DNB**	
*Rhodanobacteraceae*	0.77	*Devosia*	0.01
*Thiobacillus*	0.63	*Denitratisoma*	3.76
*Thermomonas*	0.98	*Defluviimonas*	0.03
*Thauera*	0.03	*Dechloromonas*	0.03
*Terrimonas*	1.97	*Arenimonas*	0.35
*Roseomonas*	0.009	*Bdellovibrio*	0.38
*Pseudomonas*	0.009	Total DNB	9.60
*Pedobacter*	0.38	**NOB**	
*Opitutus*	0.03	*Nitrospira*	0.11
*Nakamurella*	0.01	**AOB**	
*Mesorhizobium*	0.005	*Nitrosomonas*	0.63
*Haliangium*	0.17	**AnAOB**	
*Dokdonella*	0.05	*Candidatus Brocadia*	23.23

## Data Availability

Not applicable.
